# WHO RHN Network

**DOI:** 10.1093/eurpub/ckac129.485

**Published:** 2022-10-25

**Authors:** M Menne, A Forcellini

**Affiliations:** WHO Regions for Health Network, WHO Regional Office for Europe, Venice, Italy

## Abstract

The WHO European Office for Investment for Health and Development (Venice Office) works in the thematic areas of health equity, social and economic determinants of health, and investment for health in the context of the 2030 Agenda for Sustainable Development and the European Programme of Work. Within the WHO Venice Office, the the Regions for Health Network (RHN) works to place health and well-being high on the key political agendas, and advocates the needs of local and subnational authorities at the regional and international levels. RHN has been launched in 1993 to help regions to accelerate the delivery of improved population health, and currently has 34 members. It has developed over the decades into a forum that creates synergies between regions and stakeholders in the field of health (mutual learning); strengthens cooperation/collaboration between regional and local actors and international health institutions; increases understanding of the functioning of regional and local health systems; promotes the exchange of experience, and mutual learning. RHN positions itself at the forefront of innovative approaches and aims at becoming a cutting-edge network ready to capture and disseminate effective approaches, policies and strategies that improve population health at the regional level of governance. In past years, RHN has spearheaded collaborative efforts amongst cross-border regions, on different topics, and with technical guidance from WHO. These efforts have led to joint initiatives, publications, study trips, project collaboration and periodic and structured exchanges of experiences.

